# Supplementary figures and images for: Co-Culturing of Endothelial and Cancer Cells in a Nanofibrous Scaffold-Based Two-Layer System
Source: Int J Mol Sci. 2020 Jun 10;21(11):4128. doi: 10.3390/ijms21114128 (PMC7312426; doi:10.3390/ijms21114128)

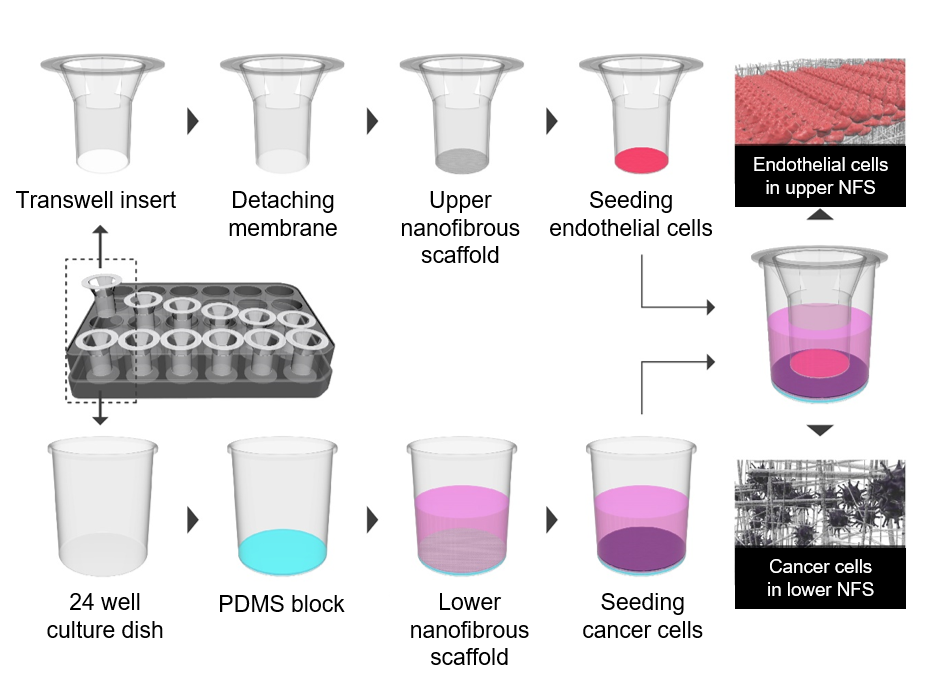

Supplement: Supplementary file 1 [file ijms-21-04128-s001.zip › Figure-S1.tif]

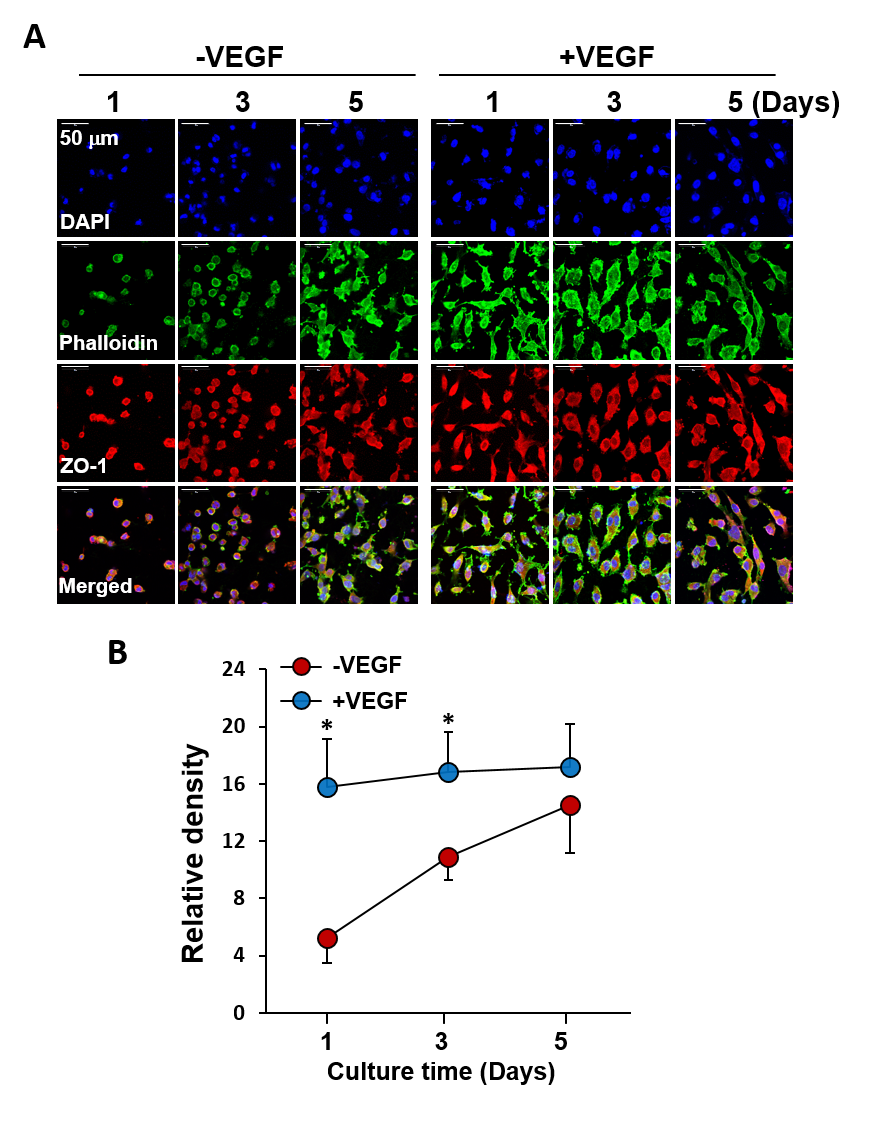

Supplement: Supplementary file 1 [file ijms-21-04128-s001.zip › Figure-S2.tif]

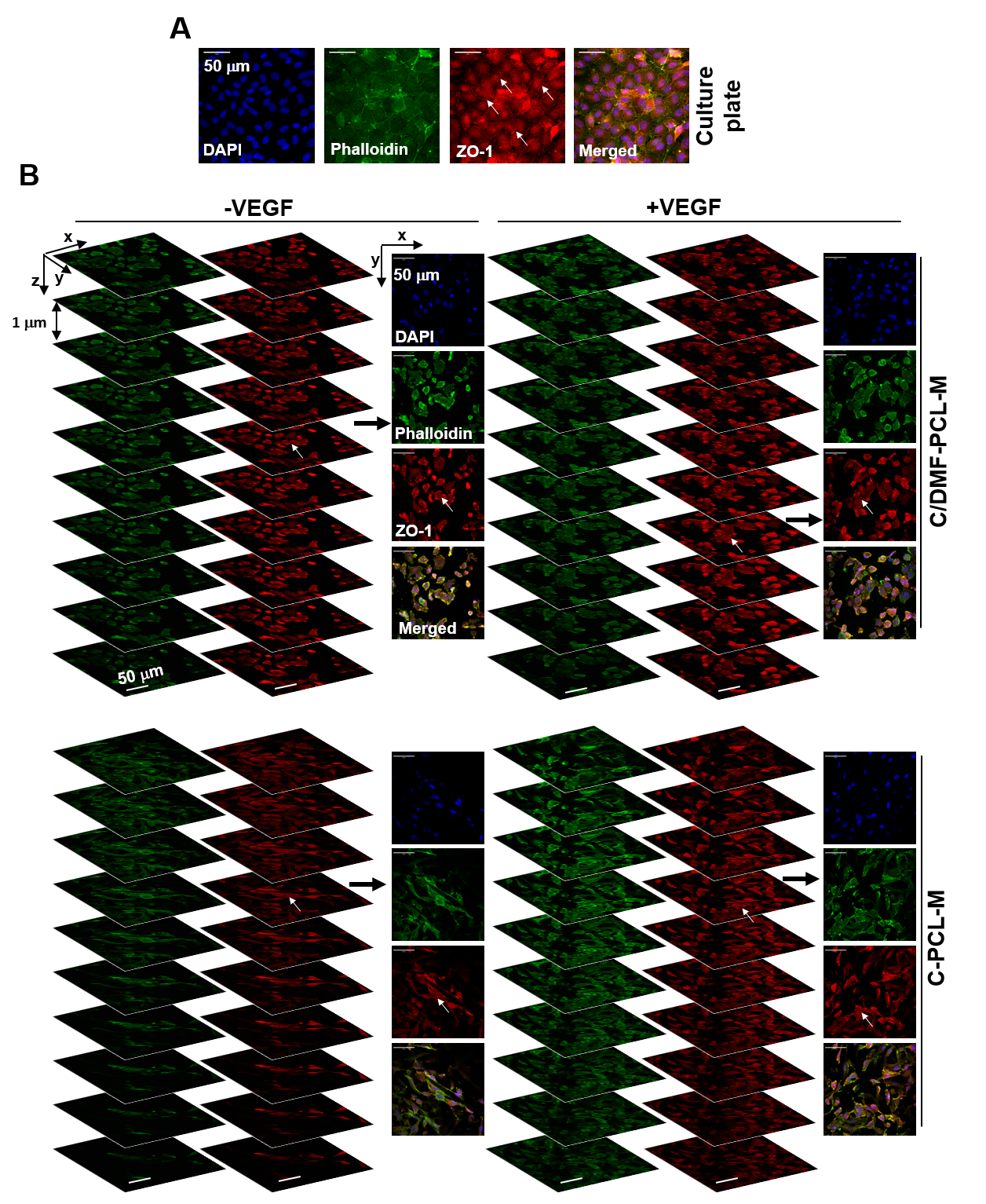

Supplement: Supplementary file 1 [file ijms-21-04128-s001.zip › Figure-S3.tif]

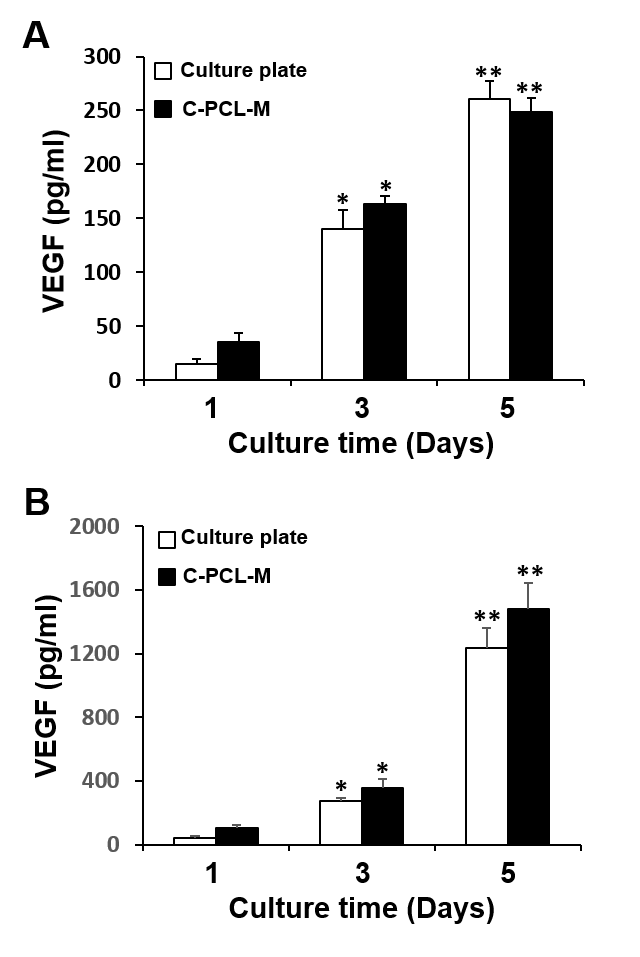

Supplement: Supplementary file 1 [file ijms-21-04128-s001.zip › Figure-S4.tif]

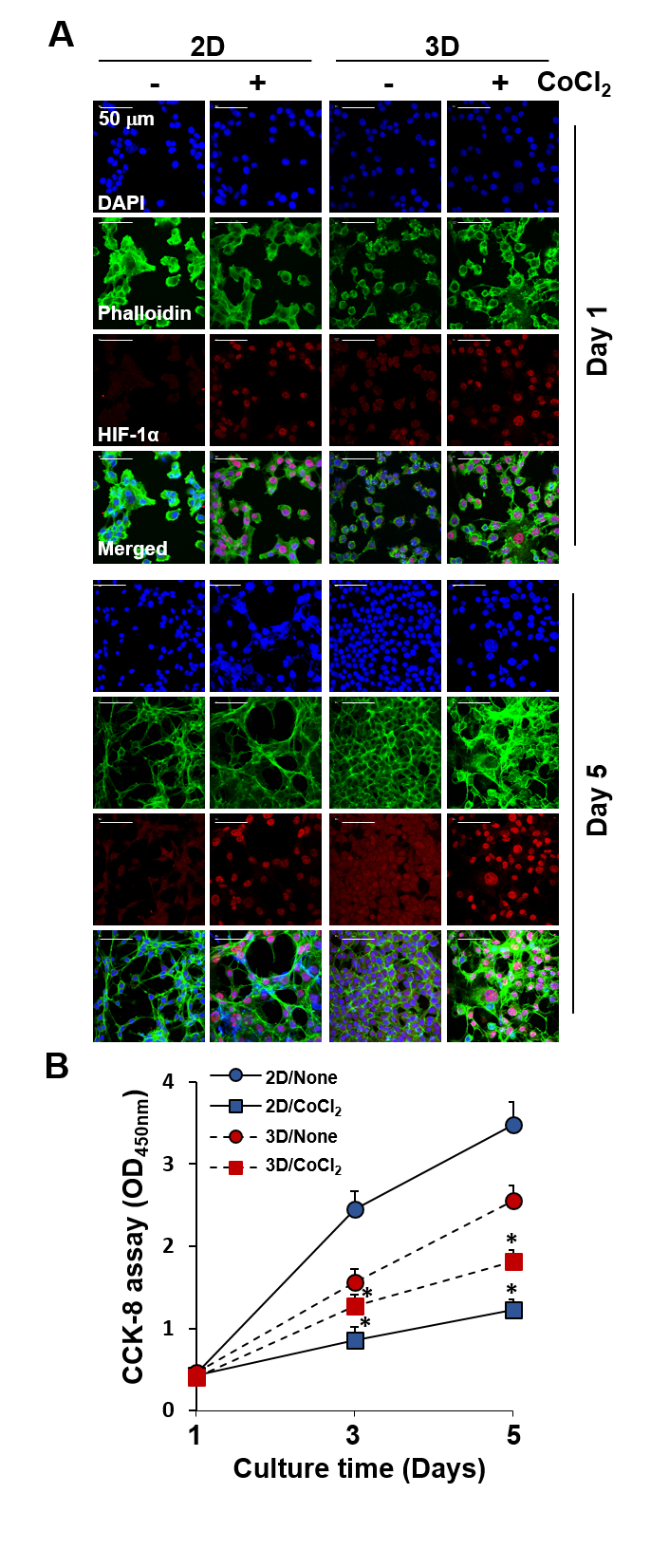

Supplement: Supplementary file 1 [file ijms-21-04128-s001.zip › Figure-S5.tif]
